# High-Frequency Local Field Potential Oscillations May Modulate Aggressive Behaviors in Mice

**DOI:** 10.3390/biology11111682

**Published:** 2022-11-21

**Authors:** Jing Yang, Yansu Liu, Yanzhu Fan, Di Shen, Jiangyan Shen, Guangzhan Fang

**Affiliations:** 1 Chengdu Institute of Biology, Chinese Academy of Sciences, No.9 Section 4, Renmin Nan Road, Chengdu 610041, China; 2 Sichuan Nursing Vocational College, No. 173 Longdu Nan Road, Chengdu 610100, China; 3 Key Laboratory of Southwest China Wildlife Resources Conservation (Ministry of Education), China West Normal University, No. 1 Shi Da Road, Nanchong 637009, China

**Keywords:** aggressive behavior, rearing pattern, local field potential, relative power spectra, mice

## Abstract

**Simple Summary:**

We investigated the dynamic patterns of neural activities related to aggressive behavior in male CD-1 mice. Our objective was to understand how rearing patterns shape aggressive behavior and which neural activities may engage in mediating aggressive behaviors in mice. We found that (1) socially isolated mice exhibited more aggression compared with cohousing mice; (2) cohousing mice exhibited significantly greater beta activities but substantially smaller gamma activities compared with the socially isolated mice; (3) gamma activities during attacks were significantly greater than that before attacks in the right ventromedial hypothalamus (VMH) of socially isolated mice. These findings suggest that rearing patterns could shape aggressive behaviors in mice and that the high frequency neural activities (beta and gamma activities) may engage in mediating these behaviors.

**Abstract:**

Aggressive behavior is one of congenital social behaviors in many species, which could be promoted by social neglect or isolation in the early stages of life. Many brain regions including the medial prefrontal cortex (mPFC), medial amygdala (MeA) and ventromedial hypothalamus (VMH) are demonstrated to relate to aggressive behavior; however, the dynamic patterns of neural activities during the occurrence of this behavior remain unclear. In this study, 21-day-old male CD-1 mice were reared in social isolation conditions and cohousing conditions for two weeks. Aggressive behaviors of each subject were estimated by the resident–intruder test. Simultaneously, the local field potentials of mPFC, MeA and VMH were recorded for exploring differences in the relative power spectra of different oscillations when aggressive behaviors occurred. The results showed that the following: (1) Compared with the cohousing mice, the socially isolated mice exhibited more aggression. (2) Regardless of “time condition” (pre-, during- and post- attack), the relative power spectra of beta band in the cohousing mice were significantly greater than those in the socially isolated mice, and inversely, the relative power spectra of gamma band in the cohousing mice were significantly smaller than those in the socially isolated mice. (3) The bilateral mPFC exhibited significantly smaller beta power spectra but greater gamma power spectra compared with other brain areas regardless of rearing patterns. (4) For the right VMH of the socially isolated mice, the relative power spectra of the gamma band during attacks were significantly greater than those before attack. These results suggest that aggressive behaviors in mice could be shaped by rearing patterns and that high-frequency oscillations (beta and gamma bands) may engage in mediating aggressive behaviors in mice.

## 1. Introduction

Aggressive behavior refers to any form of behaviors that intentionally harm another organism that is unwilling to accept it [1]. This behavior is an adaptive social behavior with which organisms can defend resources and territories in complex environments. Social isolation experienced in early life would increase aggressive behavior [2] and has an adverse effect on brain functions in animals [3,4,5]. For instance, compared with rhesus macaques (*Macaca mulatta*) that experienced competent maternal care, maltreated animals show longer reaction times to social threats, suggesting that different postnatal experiences and early biobehavioral mechanisms regulate the development of emotional attention biases during adolescence [6]. Similarly, experiencing social neglect or isolation at the early stage in humans can harm their abilities in many aspects, such as cognitive deficits, anxiety susceptibility, and increased aggressive behavior [7,8,9,10]. However, the relationship between increased aggressive behaviors due to social isolation and the dynamics of neural oscillations in the brain remains unclear.

Mice with isolation-induced aggressive behaviors are ideal animal models of offensive aggression with strong predictive validities toward human aggression [11,12]. Specifically, the most potent effects of isolation-rearing on behavioral measures in rodents occur during a critical phase from weaning to early adulthood [13]. The aggressive behavior processes of rodents generally include approaching, sniffing, biting, fighting, on-top and chasing and may be accompanied by rapid tail rattles and teeth chatter [14]. Rodents rely on olfactory cues to trigger attacks, during which olfactory information from the accessory olfactory bulb is directly transmitted to the medial amygdala (MeA), and then it is further processed and relayed to hypothalamic areas that control innate emotional, reproductive and social behaviors [15,16,17]. It has been demonstrated that most of c-fos positive MeA neurons induced by attacks are GABAergic, and the optogenetic activation of these neurons can trigger strong attacks towards gonadally intact male intruders, castrated males, females and even a toy mouse [18]. The hypothalamus is a highly conserved limbic structure related to aggression, and the ventromedial hypothalamus (VMH) serves as a key region for controlling inter-male aggression [19]. Specifically, the optogenetic or chemogenetic activation of the ventrolateral subregion of VMH (VMHvl) neurons elicits acute attacks toward males, females and even inanimate objects, while inhibition of VMHvl neurons reduces spontaneous attacks [19,20,21]. Consistent with this, a subpopulation of VMHvl neurons, tuned to both inter-male distance and animal’s velocity during attack, responds maximally during the investigation and attack of conspecific male mice and during the investigation of a source of male mouse urine [22]. The prefrontal cortex (PFC) is associated with high-level cognitive and executive functions and is generally involved in the control of the generation of overt behavioral actions or behavioral states [18]. The optogenetic activation of the principal pyramidal excitatory neurons in the medial PFC (mPFC) can effectively suppress the initiation and execution of inter-male aggression in mice; on the contrary, the optogenetic silence of mPFC neurons can cause an escalation of aggressive behaviors [23]. Therefore, mPFC is responsible for inhibiting the activities of neural circuits that strictly control the execution of attacks [24]. Accordingly, multiple brain regions engage in the initiation and execution of aggressive behaviors; however, it is still unclear how brain oscillations dynamically characterize the occurrence of aggressive behaviors.

The local field potential (LFP) is a summation signal of excitatory and inhibitory dendritic potentials from a large number of neurons in the neighborhood of the recording site [25]. Various oscillations in LFPs that originate from various cortical and subcortical structures contribute to different brain functions [26]. These periodic rhythmic oscillations in different frequency bands mainly consist of delta (1.5–4 Hz), theta (4–8 Hz), alpha (8–12 Hz), beta (12–30 Hz) and gamma (30–80 Hz) oscillations [27], which act as universal operators or codes of brain functional activity and have multifold functions [28]. In particular, delta oscillations may link to motivation and decision making [28]; theta oscillations may relate to learning and memory [29]; alpha oscillations may reflect an idling state of cortical networks and working memory [26]; beta oscillations may link to movement, cognition and olfactory processing [30,31], while gamma oscillations may relate to attention, working memory, sensory processing, action selection, movement initiation and decision making [32,33,34,35]. Each oscillation may involve the realization of multiple brain functions, and a given brain function also requires the participation of various oscillations [28]. Therefore, the brain often uses various oscillations with different frequencies to coordinate various sensory and cognitive tasks.

In order to explore the relationship between increased aggressive behaviors due to social isolation and the dynamics of neural oscillations, male CD-1 mice were randomly assigned to be housed alone or with four same-sex cagemates after weaning (i.e., on postnatal day 21) for two weeks. The aggressive behaviors of each mouse were estimated by the resident–intruder test. The LFPs of mPFC, MeA and VMH were recorded before, during and after attacks induced by an intruder. The relative power spectra were calculated for each band in order to estimate the relationship between the dynamics of LFP oscillations and aggressive behaviors. We predicted the following: (1) compared with the cohousing mice, the socially isolated mice will exhibit increased aggressive behaviors with more attacks, longer duration of attacks and/or shorter latency to the first attack; (2) the activity patterns of some LFPs oscillations will be modulated by different rearing conditions; (3) gamma oscillations will elevate during attack because of its correlation with action selection and movement initiation [32,34].

## 2. Materials and Methods

### 2.1. Animals

The 21-day-old male CD-1 mice (n = 40) were obtained from Chengdu Dossy Experimental Animals Co., Ltd. The mice were randomly divided into two groups: the cohousing group with five mice per cage and social isolation group with one mouse per cage. In addition, male C57BL/6J mice (6–8 weeks old, n = 40) obtained from the same company were housed with five mice per cage and used as intruders because of their relatively lower aggressive levels compared with male CD-1 mice [36]. During the experiments, CD-1 mice were slightly heavier than C57BL/6J mice so that CD-1 mice can retain relatively high aggressive levels and C57BL/6J mice can be protected from serious physical wounds. Each C57BL/6J mouse was involved in four trials over four days (three trials of resident–intruder test over three consecutive days for assessing the aggressive levels of the male CD-1 mice and one trial of the resident–intruder test during electrophysiological recording). Each resident faced a different intruder every day in order to prevent reducing aggressiveness because of the smell of the same intruder. All animals were raised in the same room using an individually ventilated cage system with 200 m^3^/h of ventilatory capacity (ZJ-4, Fengshi, Suzhou, China) and with food and water provided *ad libitum*. The system contained 112 cages (0.325 × 0.210 × 0.180 = 0.0122 m^3^ of volume per cage) that were located at two different racks. The ventilation rate was set to 30 per hour. The cages raising CD-1 and C57BL/6J mice were placed on different racks and kept away from each other by strain. Thus, the effects of both visual and odor cues between the two strains could be avoided. The cages were placed in a room under controlled temperature (22 ± 1 °C) and humidity (60 ± 10%), and they were maintained on a 12:12 light–dark cycle (lights on at 8:00 with about 10 lux). The subjects, i.e., CD-1 mice, were kept in the corresponding conditions for two weeks before further behavioral experiments. The timeline of experiments started with a three-day resident–intruder test, followed by one day for surgery, at least five days for recovery and one more day for electrophysiological recording. All experiments were performed during the animals’ dark phase under a dim red light (<2 lux).

### 2.2. Resident–Intruder Test

Aggressive abilities were measured by observing the responses of a resident male mouse toward a novel male mouse (intruder) [37]. At least 15 min before testing, the residents (CD-1) and intruders (C57BL/6J) within their own cages were moved into two identical electromagnetically shielded and soundproof chambers, respectively, and were left undisturbed. In the test stage, each CD-1 mouse in his own cage was exposed to an unfamiliar intruder for 180 s daily over three consecutive days (for the cohousing mice, his cagemates would be moved to a new cage before the test). Attacks were defined by a suite of intense actions initiated by the resident toward the intruder, which included bites, boxes, on-top and fast locomotion episodes between such behaviors [36]. Moreover, the chase was defined as a quick follow initiated by the resident toward the intruder. For social interactions, sniff was defined as nose contact made by the resident to any part of the intruder’s body. Play fighting is a common activity for the juveniles of many species of mammals, and some previous studies classified biting the intruder’s nape (which always results in the intruder’s rotation to a fully supine position) into play fighting [38,39,40]. All bites initiated by CD-1 mice to any body part of C57BL/6J mice usually lead to the latter being moved away or a rotation to a fully supine position, which suggests that biting the nape is similar to biting other body parts for the C57BL/6J mouse used in the present study. Accordingly, we did not separate biting nape behaviors from aggression, as many other studies did [41,42,43]. The animals’ behaviors were recorded using two synchronized cameras placed on one side of the cage and on the top of the cage (C930e, Logitech, Suzhou, China). A free and versatile open-source event-logging software (BORIS) was used for further video analysis [44]. Specifically, the video was manually analyzed frame by frame by a single experimenter who was blind to the grouping of data. The onset and ending of each behavior were marked using BORIS for further analysis. The total number of each type of behavior (attack, chase and sniff), latency to the first attack and cumulative durations of attack and chase were measured. If the subject had no aggressive behavior in the three consecutive days, its behavioral parameters related to attack had been excluded for further statistical analyses.

### 2.3. Surgery

**Figure 1:**
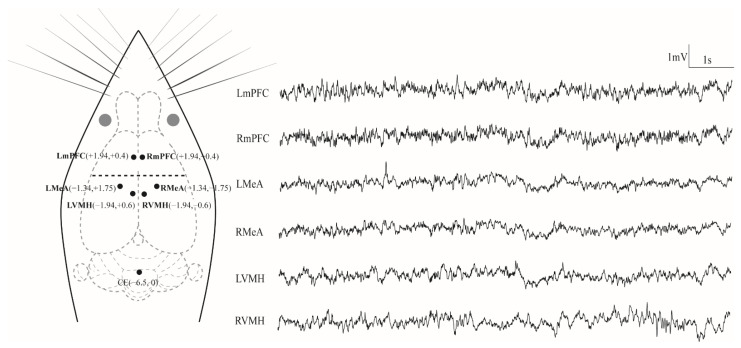
Electrode placements and 10 s of typical LFP tracings for each channel. LmPFC, RmPFC, LMeA, RMeA, LVMH and RVMH represent the left and right medial prefrontal cortex, medial amygdala and ventromedial hypothalamus, respectively, while CE denotes the reference electrode implanted above the cerebellum. The grey circles denote the eyes of the animal.

After completing the resident–intruder tests, all CD-1 mice were further conducted for the electrophysiological experiments. The subject was placed in an induction chamber, which was connected to an anesthesia machine for small animals (R500IE, RWD, Shenzhen, China) for induction anesthesia with 2–3% isoflurane. After achieving the effects of anesthesia, the anesthetized animal was transferred from the induction chamber onto the mouse adaptor and head-fixed in a mouse stereotaxic apparatus (51603, Stoelting, Chicago, IL, USA). The animal breathed via an anesthesia mask that supplies a constant flow of anesthetic gas (approximately 1.5% isoflurane) to keep the animal safely anesthetized during surgery. Any excess anesthetic vapor is scavenged away from the animal via the outer tube of the mask and drawn through a canister of activated charcoal, which absorbs the excess anesthetic gas. According to the stereotaxic atlas of mice [45], six LFP recording electrodes composed of a single surface-insulated stainless-steel wire (0.1 mm in diameter) were implanted inside the target regions: bilateral mPFC (anteroposterior (AP) +1.94 mm, mediolateral (ML) ±0.4 mm and dorsoventral (DV) −2.1 mm from bregma), bilateral MeA (AP −1.34 mm, ML ±1.75 mm and DV −5.4 mm from bregma) and bilateral VMH (AP −1.94, ML ±0.6 mm and DV −5.8 mm from bregma; Figure 1). For mPFC and MeA, the coordinates used for each electrode were located on the inner granular layer (IV). Because the LFP signals measured by an electrode come from neurons within a radius of about 200–300 μm [46], the activities of both the outer pyramidal layer (III) and inner pyramidal layer (V) can be recorded. The reference electrode (CE) was implanted above the cerebellum. In order to fix the electrodes, four miniature stainless-steel screws (0.8 mm in diameter) were implanted into the skull for encompassing the six recording electrodes. All electrodes were fixed on the skull with dental acrylics. Before performing further experiments, the subjects were permitted to recover for at least five days.

### 2.4. Electrophysiological Recording and Data Processing

A CD-1 mouse was placed in his home-cage and was connected to a signal acquisition system (RM6280C; Chengdu, China) for 15 min habituation. A low-pass filter with a cutoff frequency at 500 Hz was used for LFP signal recordings, while the notch filter was set to eliminate possible interferences at 50 Hz and the sampling frequency was set to 1000 Hz. Both LFP and behavioral data were recorded when the intruder was placed in the resident’s home cage, and free social interactions were permitted for 180 s. The experimenter presses a button when the intruder is introduced so that a red LED outside of the home-cage lights up and a trigger is sent to the signal acquisition system for synchronizing the behavioral and LFP data.

In order to compare the differences in the LFP power spectra between the two groups, only LFP data acquired from nine socially isolated mice with more than three attacks and thirteen cohousing mice without any attacks were conducted for further data processing. The LFP data were filtered offline using a band-pass filter of 0.5–80 Hz. We used the onset time and the ending time of each attack to find pre-, during- and post-attack periods for both behavioral and LFP data via the synchronizing timestamp between these two types of data. Then, two consecutive artifact-free 1 s segments were extracted for pre-, during- and post-attacks for each attack (Figure S1). For each cohousing mouse, each of the three timestamps was selected randomly for each minute and regarded as the onset of an attack. Using Welch’s method with a Hanning window (50% overlap) and 0.5 Hz resolution, the power spectral densities were calculated for each segment, each brain region and each time condition (i.e., pre-, during- and post-attack) (Figure S2). Compared with absolute power, relative power (band power/total power across bands) is more suitable for comparisons between different brain regions. Accordingly, the relative power spectra were acquired from the corresponding power spectral densities for each band, i.e., delta (1.5–5 Hz), theta (5–8 Hz), alpha (8–12 Hz), beta (12–30 Hz) and gamma (30–80 Hz). For each subject, the relative power spectra were averaged across the two segments and different attack bouts for each brain region, each time condition and each band. Furthermore, we use time–frequency analysis to characterize changes in the spectral content of the data for detecting the instantaneous perturbations of each band. Specifically, each attack was considered as an event and the two-consecutive artifact-free 1 s segments were averaged for each brain region, each time condition and each subject. The average waveforms were further averaged across the socially isolated mice and the cohousing mice, respectively. The event-related spectral perturbations were calculated using EEGLAB toolbox with default parameters [47].

### 2.5. Histology

Upon the completion of LFP and behavioral recordings, we used a program-controlled stimulator (YC-2, Chengyi, Chengdu, China) to conduct current damage to each brain area where the electrode was implanted with a current intensity of 0.4 mA for 8 s for identifying the locations of the electrode tips. Then, the mouse was deeply anesthetized and transcardially perfused with cold phosphate-buffered saline (PBS) followed by fixation with cold 4% paraformaldehyde (PFA) in a PBS solution. The brain was removed and post-fixed in a 4% PFA solution overnight at 4 °C and cryoprotected (30% sucrose in PBS, 4 °C). The frozen brain was sectioned into 30 μm-thick coronal sections using a cryostat (CM1850, Leica, Nussloch, Germany) and collected in PBS. The coronal sections stained with DAPI were mounted on slides in order to visualize and image the lesion location using a digital slice scanner (Pannoramic DESK, 3DHISTECH, Budapest, Hungary). The recording sites were verified histologically by referencing the mouse brain atlas [45] and excluded data at non-target sites (Figure S3).

### 2.6. Statistical Analyses

For behavioral parameters and LFP relative power spectra, the normality of distributions and the homogeneity of the variance of values were tested using the Shapiro–Wilk test and Levene’s test, respectively. For the number of sniffs, two-way repeated-measures ANOVA was carried out with the factors “group” (the cohousing group and social isolation group) and “day” (the three consecutive days for resident–intruder test, i.e., Day 1, Day 2 and Day 3). Because other behavioral parameters failed to meet the statistical assumptions, the Mann–Whitney U test was used to test the difference between the cohousing group and social isolation group for each parameter. For the LFP relative power spectra, three-way repeated-measures ANOVA was carried out with the factors “group”, “brain area” (the six brain regions) and “time condition” (pre-, during- and post- attack). Both main effects and interactions were examined. Moreover, if the interaction was significant, the simple or simple–simple effects analysis was applied. If ANOVA returned a significant difference, multiple comparisons were conducted using the least significant difference (LSD) test. Greenhouse–Geisser epsilon (ε) values were employed if the assumption of sphericity was violated. The effect size was determined with partial *η*^2^ (partial *η*^2^ = 0.20 is set as small, 0.50 is set as medium, and 0.80 is set as a large effect size). Statistical analyses were performed using SPSS software (release 23.0), *p*-values were marked statistically significant as follows: * *p* < 0.05 and ** *p* < 0.001.

## 3. Results

### 3.1. The Results of Aggressive Behaviors

**Figure 2:**
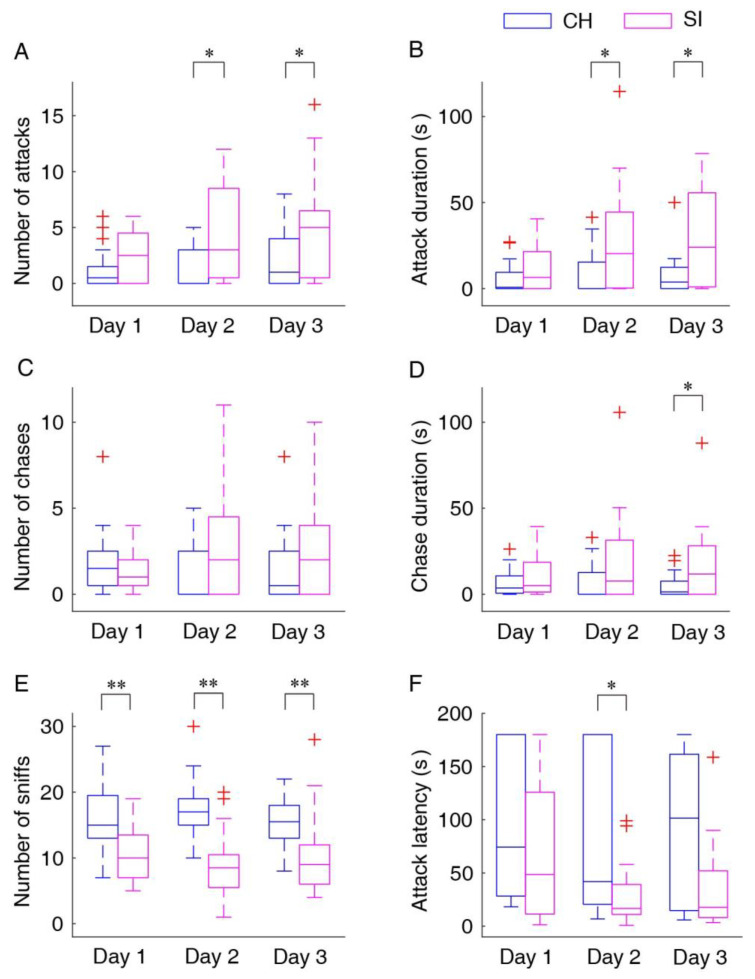
Statistical results for different behavioral parameters: number of attacks (**A**), attack duration (**B**), number of chases (**C**), chase duration (**D**), number of sniffs (**E**) and attack latency (**F**). Data points beyond the whiskers are displayed using red +. SI, the socially isolated mice; CH, the cohousing mice; Day 1–3, the three consecutive days for the resident–intruder test. * *p* < 0.05; ** *p* < 0.001.

Although the difference for the first day did not reach statistical significance, the number of attacks in the socially isolated mice was significantly greater than those in the cohousing mice (*U* = 143.5, *N* = 40, *p* = 0.11 for Day 1; *U* = 116.5, *N* = 40, *p* = 0.019 for Day 2; *U* = 121, *N* = 40, *p* = 0.029 for Day 3; Figure 2A). The socially isolated mice spent significantly more time on aggressive behaviors compared with the cohousing mice, although the difference for the first day did not reach statistical significance (*U* = 153, *N* = 40, *p* = 0.186 for Day 1; *U* = 115.5, *N* = 40, *p* = 0.018 for Day 2; *U* = 118.5, *N* = 40, *p* = 0.024 for Day 3; Figure 2B). Meanwhile, there was no significant difference in the number of chases between the two groups (Figure 2C), but the socially isolated mice spent more time on chases although the significant difference only exhibited on the last day (*U* = 130, *N* = 40, *p* = 0.049 for Day 3; Figure 2D). For the number of sniffs, the main effect was significant for the factor “group” (*F*_1,38_ = 20.905; *p* < 0.001, partial *η*^2^ = 0.44), and the number of sniffs in the cohousing mice was significantly greater than that in the socially isolated mice (*p* < 0.001; Figure 2E). In addition, the latencies to the first attack were significantly shorter in the socially isolated mice than those in the cohousing mice although a significant difference was only exhibited on the second day (*U* = 57, *N* = 29, *p* = 0.036 for Day 2; Figure 2F).

### 3.2. Relative Power Spectra for Each EEG Band

**Figure 3:**
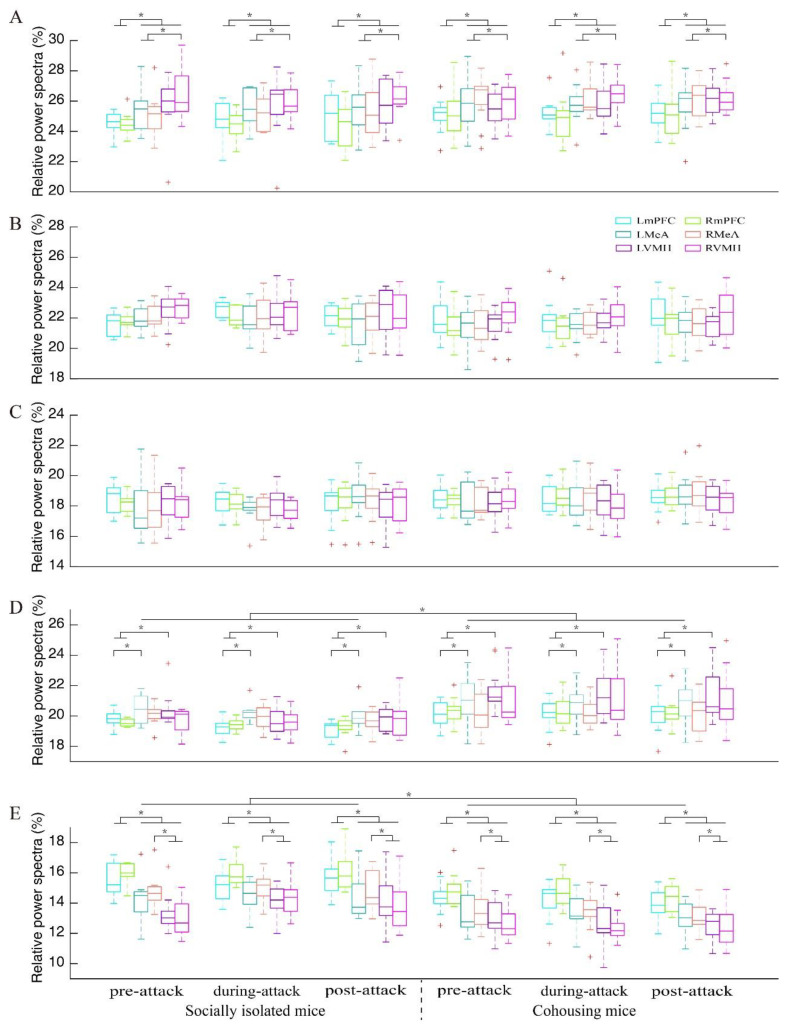
Relative power spectra for each brain area and each time condition for delta (**A**), theta (**B**), alpha (**C**), beta (**D**) and gamma (**E**) bands in the socially isolated mice and cohousing mice. Data points beyond the whiskers are displayed using red +. LmPFC and RmPFC, the left and right medial prefrontal cortex; LMeA and RMeA, the left and right medial amygdala; LVMH and RVMH, the left and right ventromedial hypothalamus. * *p* < 0.05.

Time-frequency analysis showed that attack behaviors were accompanied by increased neural activities, especially in the gamma band, in all brain areas (Figure S4). For the delta band, there was a significant main effect for the factor “brain area” (*F*_5,100_ = 9.091; *p* < 0.001, partial *η*^2^ = 0.312) but not the factors “group” (*F*_1,20_ = 1.283; *p* = 0.271, partial *η*^2^ = 0.060) and “time condition” (*F*_2,40_ = 0.083; *p* = 0.920, partial *η*^2^ = 0.004; Figure 3A and Table 1). The power spectra of the bilateral VMH and MeA were significantly greater than those of the bilateral mPFC (all *p* ≤ 0.027), while the power spectra of the right VMH was significantly greater than those of the bilateral MeA (all *p* ≤ 0.049).

For the theta band, there was no significant main effect for the factors “brain area” (*F*_5,100_ = 2.322; *p* = 0.079, partial *η*^2^ = 0.104), “group” (*F*_1,20_ = 1.733; *p* = 0.203, partial *η*^2^ = 0.080) and “time condition” (*F*_2,40_ = 0.104; *p* = 0.850, partial *η*^2^ = 0.005; Figure 3B and Table 1). Similarly, there was no significant main effect for the factors “brain area” (*F*_5,100_ = 0.703; *p* = 0.531, partial *η*^2^ = 0.034), “group” (*F*_1,20_ = 1.334; *p* = 0.262, partial *η*^2^ = 0.063) and “time condition” (*F*_2,40_ = 1.073; *p* = 0.352, partial *η*^2^ = 0.051) for the alpha band (Figure 3C and Table 1).

For the beta band, there were significant main effects for the factors “brain area” (*F*_5,100_ = 5.640; *p =* 0.001, partial *η*^2^ = 0.220) and “group” (*F*_1,20_ = 9.582; *p* = 0.006, partial *η*^2^ = 0.324) but not “time condition” (*F*_2,40_ = 1.674; *p* = 0.200, partial *η*^2^ = 0.077; Figure 3D and Table 1). The power spectra of the left VMH were significantly greater than those of the bilateral mPFC (all *p* ≤ 0.003), while the beta power spectra of the left MeA were significantly greater than that of the left mPFC (*p* = 0.042). In addition, the power spectra of the cohousing mice were significantly greater than those of the socially isolated mice.

For the gamma band, there were significant main effects for the factors “brain area” (*F*_5,100_ = 31.273; *p* < 0.001, partial *η*^2^ = 0.610) and “group” (*F*_1,20_ = 15.553; *p* = 0.001, partial *η*^2^ = 0.437) but not “time condition” (*F*_2,40_ = 0.450; *p* = 0.641, partial *η*^2^ = 0.022; Figure 3E and Table 1). Moreover, the interaction among the three factors was significant (*F*_1,20_ = 2.734; *p* = 0.004, partial *η*^2^ = 0.120; Table 1). The simple–simple effects analysis showed that the power spectra in the right VMH for “during-attack” was significantly greater than that for “pre-attack” in the socially isolated mice (*p* = 0.005; Table 2 and Figure S4). In general, the power spectra of the bilateral mPFC were significantly greater than those for other brain areas, regardless of “group” and “time condition” (all *p* < 0.001; Table 2). The gamma power spectra in the socially isolated mice were significantly greater than those in the cohousing mice for most brain area during pre-, during- and post- attack (all *p* ≤ 0.040; Table 2).

## 4. Discussion

### 4.1. Social Isolation Promoted Aggressive Behaviors

Early social isolation is a main factor that induces various emotional, behavioral and cognitive abnormalities in both animals and humans [10,48]. For example, social isolation can induce increased attack frequency and shorten attack latencies in male mice; that is, resident mice usually exhibit more aggressive behaviors against intruder mice compared with cohousing ones [14,42]. Consistent with this, the present results showed that the socially isolated mice were more aggressive compared with the cohousing ones, and they manifested more attacks and chases, a longer duration of attacks and shorter latencies for the first attack; on the contrary, the cohousing mice showed less aggression and more sniffs. The behavioral differences between the two groups may result from different rearing patterns. Unlike the socially isolated mice, the cohousing mice were kept in a social rearing pattern during which the mice could receive all kinds of cues (including visual, auditory and olfactory ones) from companions to maintain normal social interaction and emotional relationships [49]. Consequently, the cohousing mice sniffed the intruder mice more frequently and exhibited increased exploratory activities compared with the socially isolated mice when the intruder was introduced.

Moreover, the present results showed that there was no differences in the amount of sniffing across the three consecutive test days for both the socially isolated mice and cohousing mice, and this is inconsistent with a previous study that showed that the amount of sniffing of the cohousing mice gradually decreased with trials conducted on a test day [50]. This divergence might result from different experimental designs. Kercmar and her colleagues used a standard social recognition test to explore the differences in sniffing behaviors between the socially isolated mice and cohousing mice, during which all mice were exposed eight times at 1 min each to the same stimulus mouse followed by the ninth test with a new stimulus mouse. They found a pattern of social habituation in the cohousing mice but not the socially isolated ones, where time spent on sniffing the stimulus mice decreased significantly from trials 1 to 8 [50]. On the contrary, a resident–intruder test was used in the present study, during which each CD-1 mouse in his own cage was exposed to an unfamiliar intruder for 180 s daily over three consecutive days; thus, similar social habituation could not be found.

### 4.2. High-Frequency EEG Oscillations May Modulate Aggressive Behaviors in Mice

Neuronal oscillations with various frequency bands are proposed as important factors for neuron assembly synchronization and binding as well as plasticity [51]. They act as universal operators or codes of brain functional activities and, thus, engage multiple physiological functions [28]. For example, beta oscillations engage every part of the olfactory system and exhibit behavior that is reliably coherent across different brain regions during odor processing [31]. Perceptual constancy requires the brain to maintain a stable representation of sensory inputs [52,53]; it seems reasonable to speculate that beta oscillation in various brain regions might involve encoding and delivering the associative value of a given odor. One of the most critical upstream sensory pathways mediating the choice of nearly all social behaviors including aggression is olfaction in rodents [54] and that the olfactory information ascends mainly ipsilaterally from each nostril to the primary olfactory cortex (piriform cortex, PC) in the same hemisphere in mammals [55]. Moreover, MeA receives direct chemosensory inputs from the accessory olfactory bulb [56] and orchestrates various behavioral outputs via brain-wide projections [57]. The current results show that the beta power in the left MeA and left VMH were greater than those in the bilateral mPFC when the intruder was introduced, consistent with the fact that the left hemisphere is primarily involved during olfactory perception in rodents [58,59,60,61]. In the olfactory system, the activity in PC is thought to determine odor identities [52,53]. Specifically, the left anterior PC displays significantly greater beta oscillations than the right counterpart during odor discrimination learning [60,61]. Compared with the socially isolated mice, the cohousing mice could use all kinds of cues for maintaining emotional relationships and normal social interactions [49]. Consequently, it is expected that the cohousing mice would recruit more brain resources for social interaction when the intruder was introduced. Congruent with this, we found that the cohousing mice sniffed the intruder mice more frequently and exhibited increased exploratory activities compared with the socially isolated mice, and that the beta power in the cohousing mice was significantly greater than that in the socially isolated ones.

Gamma oscillations can provide an ideal mechanism to coordinate precise neural coding within and across brain structures because of their fast temporal dynamics [32]; thus, these oscillations are tied to a diverse array of neural functions, including attention, working memory, sensory processing, action selection, movement initiation and decision making [32,33,34]. Interestingly, compared with women, men usually exhibit more aggressive behaviors and greater gamma signals in the right ventrolateral frontal cortex evoked by aggressive videos [62]. Consistent with this, the present results show that the socially isolated mice exhibited significantly greater gamma activities compared with the cohousing mice, and the gamma power spectra in the right VMH of the socially isolated mice during attack was significantly greater than that during pre-attack periods. Because VMH serves as a key region to control inter-male aggression [19], the elevated gamma activities in VMH of the socially isolated mice might be an indicator of attack initiations. Consistent with this, the present results showed that the onset of attack was accompanied with increased spectral perturbations in the gamma band.

The current results show that the power spectra of gamma oscillations in the bilateral mPFC were significantly greater than those in other brain regions, in concert with the idea that mPFC plays a key role in decision making and goal-directed behavior [63,64]. However, this is not in accord with the clarification that mPFC is responsible for inhibiting the activities of neural circuits that strictly control the execution of attacks [23]. In fact, the optogenetic activation (472 nm blue light with 20 Hz) of the mPFC excitatory neurons can effectively suppress the initiation and execution of inter-male aggression in mice; on the contrary, the optogenetic silence (589 nm yellow light with 20 Hz) of mPFC neurons causes an escalation of aggressive behaviors [23]. However, optogenetic activations with an 80 Hz 472 nm blue light of the mPFC excitatory neurons could increase aggressive behaviors [23]. Because a given frequency of optogenetic stimulus in a specific brain region would cause neural oscillations entrainment with specific frequency [65,66], further studies will be needed to determine the relationship across aggressive behavior, activation frequencies and specific neural oscillations.

## 5. Conclusions

In summary, we found that the following: (1) compared with the cohousing mice, the socially isolated mice exhibited more aggression; (2) compared with the socially isolated mice, the cohousing mice exhibited significantly greater beta activities but substantially smaller gamma activities regardless of “time condition” (pre-, during- and post- attack); (3) generally, the beta oscillations in the left MeA and left VMH were greater than those in the bilateral mPFC, while the gamma oscillations in the bilateral mPFC were significantly greater than those in other brain regions regardless of rearing patterns. In addition, the gamma band during attacks was significantly greater than that before attacks in the right VMH of the socially isolated mice. These findings suggest that aggressive behaviors in mice could be shaped by rearing patterns, and that the high frequency oscillations (beta and gamma bands) may engage in mediating aggressive behaviors in mice. However, future studies are needed for a comprehensive understanding of the relationship between the initiation of aggressive behavior and beta and/or gamma oscillations and whether MeA-VMH or mPFC-VMH neural activities synchronize in beta or gamma frequencies. Since the social isolation of rodents after weaning produces permanent neurochemical and structural alterations in various brain areas, including mPFC, MeA and VMH [2,67,68,69], how these alterations impact aggressive behaviors in mice require further elucidation.

## Figures and Tables

**Table 1:** Results of AVOVA for the factors “group”, “time condition” and “brain area” for the five EEG bands.

For the Relative Power Spectra (1, 20)(2, 40)(5, 100) ^a^				
	Group	Time Condition	Brain Area	Group × Time Condition × Brain Area
**Delta**				
*F*	1.283	0.083	9.091	1.105
*P*	0.271	0.920	0.000 **	0.360
*ε*	NA	0.818	0.727	NA
*η^2^*	0.060	0.004	0.312	0.052
LSD	NA	NA	LMeA, RMeA, LVMH, RVMH > LmPFC, RmPFC RVMH > LMeA, RMeA	NA
**Theta**				
*F*	1.733	0.104	2.322	1.113
*P*	0.203	0.850	0.079	0.354
*ε*	NA	0.762	0.642	NA
*η^2^* LSD	0.080 NA	0.005 NA	0.104 NA	0.053 NA
**Alpha**				
*F*	1.334	1.073	0.703	0.428
*P*	0.262	0.352	0.531	0.932
*ε*	NA	0.822	0.502	NA
*η^2^* LSD	0.063 NA	0.051 NA	0.034 NA	0.021 NA
**Beta**				
*F*	9.582	1.674	5.640	0.812
*P*	0.006 *	0.200	0.001 *	0.617
*ε*	NA	0.795	0.659	NA
*η^2^*	0.324	0.077	0.220	0.039
LSD	CH > SI	NA	LVMH > LmPFC, RmPFC LMeA > LmPFC	NA
**Gamma**				
*F*	15.553	0.450	31.273	2.734
*P*	0.001 *	0.641	0.000 **	0.004 *
*ε*	NA	0.975	0.696	NA
*η^2^*	0.437	0.022	0.610	0.120
LSD	SI > CH	NA	LmPFC, RmPFC > LMeA, RMeA, LVMH, RVMH RMeA > LVMH, RVMH	See Table 2

Note: The symbols “>” denote that the relative power spectra of a given EEG band for brain areas or each group on the left side of “>” are significantly greater than those on the right side, and no significant difference exists among the corresponding conditions on the same side of “>” for each case. The superscript symbol “a” in the first line of the table denotes the degrees of freedom for the factors “group”, “time condition” and “brain area”. Abbreviations: F, the *F* value from ANOVA; Partial *η^2^*, effect size for ANOVA; *ε*, the values of epsilon of the Greenhouse–Geisser correction; LSD, least-significant difference test; LmPFC and RmPFC, the left and right medial prefrontal cortex; LMeA and RMeA, the left and right medial amygdala; LVMH and RVMH, the left and right ventromedial hypothalamus; SI, the socially isolated mice; CH, the cohousing mice; NA, not applicable. * *p* < 0.05; ** *p* < 0.001.

**Table 2:** Results of simple-simple effects analysis for the interaction among the factors “group”, “time condition” and “brain area” for the gamma band.

	*F*_(1,20)(2,19)(5,16)_^a^	*p*	Partial *η^2^*	LSD
Time|(RVMH, SI)	7.191	0.005 *	0.431	During > Pre
Brain area|(Pre, SI)	20.905	0.000 **	0.867	LmPFC, RmPFC > RMeA > LMeA, LVMH, RVMH LMeA > RVMH
Brain area|(Pre, CH)	18.507	0.000 **	0.853	LmPFC, RmPFC > LMeA, RMeA, LVMH, RVMH
Brain area|(During, SI)	10.857	0.000 **	0.772	RmPFC> LmPFC, LMeA, RMeA, LVMH, RVMH LmPFC > LMeA RMeA > LMeA, LVMH
Brain area|(During, CH)	17.330	0.000 **	0.844	RmPFC > LmPFC > LMeA, RMeA > LVMH, RVMH
Brain area|(Post, SI)	11.097	0.000 **	0.776	LmPFC, RmPFC > LMeA, RMeA, LVMH, RVMH RMeA > RVMH
Brain area|(Post, CH)	10.737	0.000 **	0.770	LmPFC, RmPFC > LMeA, RMeA, LVMH, RVMH RMeA > LVMH
Group|(LP, Pre)	7.090	0.015 *	0.262	SI > CH
Group|(LP, Post)	14.047	0.001 *	0.413	SI > CH
Group|(RP, Pre)	8.996	0.007 *	0.310	SI > CH
Group|(RP, During)	7.693	0.012 *	0.278	SI > CH
Group|(RP, Post)	13.643	0.001 *	0.406	SI > CH
Group|(LM, During)	5.169	0.034 *	0.205	SI > CH
Group|(LM, Post)	5.798	0.026 *	0.225	SI > CH
Group|(RM, Pre)	6.704	0.023 *	0.233	SI > CH
Group|(RM, During)	8.437	0.009 *	0.297	SI > CH
Group|(RM, Post)	11.679	0.003 *	0.369	SI > CH
Group|(LH, During)	7.143	0.015 *	0.263	SI > CH
Group|(LH, Post)	8.501	0.009 *	0.298	SI > CH
Group|(RH, During)	19.780	0.000 **	0.497	SI > CH
Group|(RH, Post)	4.839	0.040 *	0.195	SI > CH

Note: The symbols “>” denote that the relative power spectra of gamma band associated with “group”, “time condition” or “brain areas” on the left side of “>” are significantly greater than those on the right side, and no significant difference exists among the corresponding conditions on the same side of “>” for each case. The symbols “|” denote the conditions on the left side of “|” under the conditions on the right side of “|”. The superscript symbol “a” in the first line of the table denotes the degrees of freedom for the factors “group”, “time condition” and “brain area”, respectively. Abbreviations: F, the *F* value from ANOVA; Partial *η^2^*, effect size for ANOVA; LSD, least-significant difference test; LmPFC and RmPFC, the left and right medial prefrontal cortex; LMeA and RMeA, the left and right medial amygdala; LVMH and RVMH, the left and right ventromedial hypothalamus; SI, the socially isolated mice; CH, the cohousing mice; Pre, pre-attack period; During, attack period; Post, post-attack period; NA, not applicable. * *p* < 0.05; ** *p* < 0.001.

## Data Availability

The datasets used and/or analyzed during the current study are available from the corresponding author upon reasonable request.

## Supplementary Materials

The following supporting information can be downloaded at: https://www.mdpi.com/article/10.3390/biology11111682/s1, Figure S1: The average waveforms of local field potentials (LFP; averaged across all subjects for each group) for each brain area and each situation for the socially isolated mice (A) and the cohousing mice (B); Figure S2: The average waveforms of power spectral density (PSD) for each brain area and each situation for the socially isolated mice (A) and the cohousing mice (B); Figure S3: Verification results of electrode positions for both sides of the medial prefrontal cortex (the first row), medial amygdala (the second row), and ventromedial hypothalamus (the last row); Figure S4. Time–frequency maps of grand mean local field potential (LFP) waveforms across subjects for each brain area and each situation for the socially isolated mice (A) and the cohousing mice (B).

## Abbreviations

AP	anteroposterior
CE	reference electrode
CH	the cohousing mice
DV	dorsoventral
LFP	local field potential
LMeA and RMeA	the left and right medial amygdala
LmPFC and RmPFC	the left and right medial prefrontal cortex
LSD	least significant difference
LVMH and RVMH	the left and right ventromedial hypothalamus
MeA	medial amygdala
ML	mediolateral
mPFC	medial prefrontal cortex
NA	not applicable
PBS	phosphate-buffered saline
PC	piriform cortex
PFA	paraformaldehyde
PFC	prefrontal cortex
SI	the socially isolated mice
VMH	ventromedial hypothalamus
VMHvl	ventrolateral subregion of VMH
