# Supplementary material for: High-Frequency Local Field Potential Oscillations May Modulate Aggressive Behaviors in Mice
Source: Biology (Basel). 2022 Nov 21;11(11):1682. doi: 10.3390/biology11111682 (PMC9687601; doi:10.3390/biology11111682)
Supplement: Supplementary file 1 [file biology-11-01682-s001.zip › biology-1952717-supplementary.pdf]

Supplementary materials

High-Frequency Local Field Potential Oscillations May Modulate Aggressive Behaviors in Mice

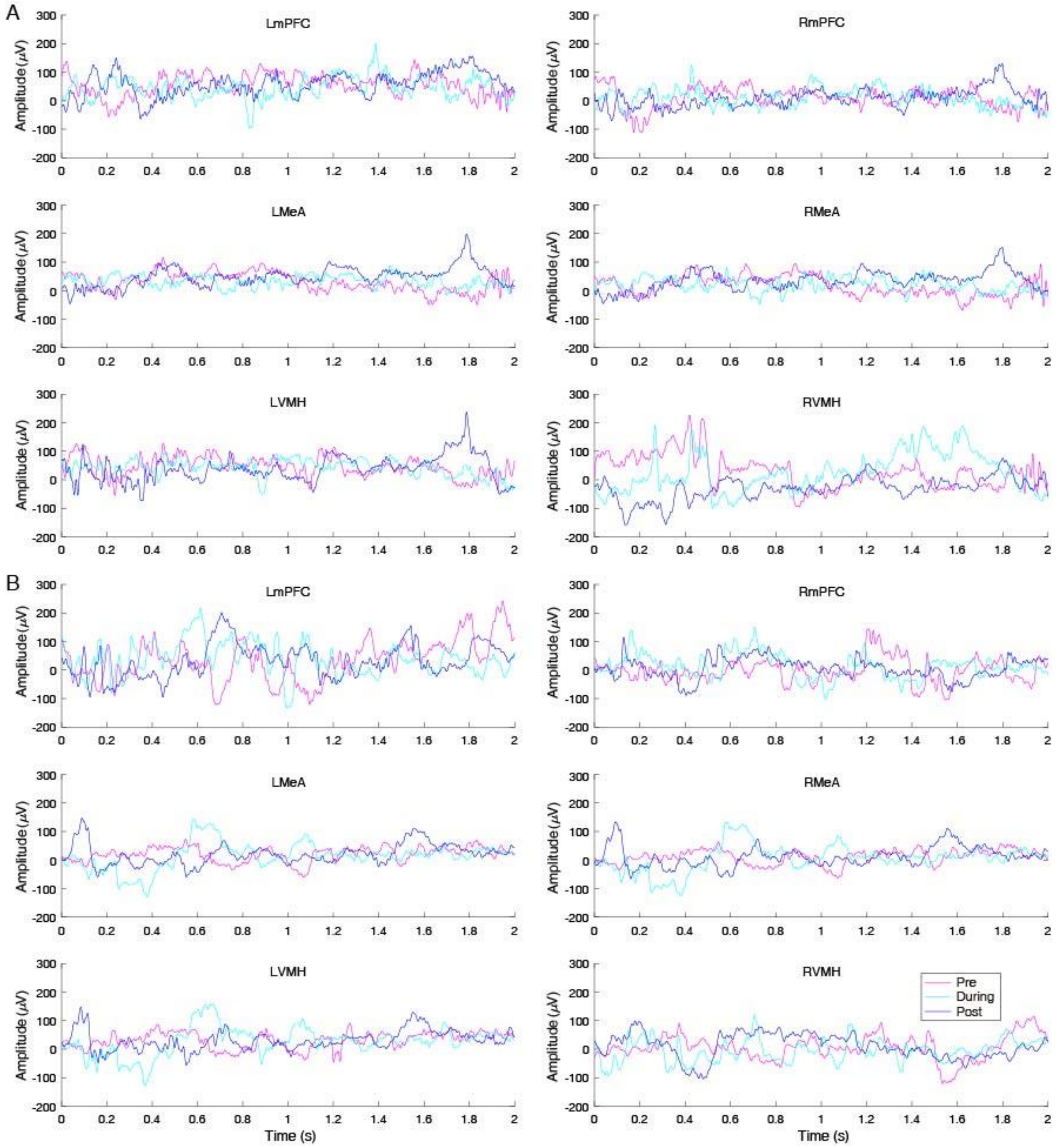

Figure S1. The average waveforms of local field potentials (LFP; averaged across all subjects for each group) for each brain area and each situation for the socially isolated mice (A) and the cohousing mice (B). LmPFC and RmPFC, the left and right medial prefrontal cortex; LMeA and RMeA, the left

and right medial amygdala; LVMH and RVMH, the left and right ventromedial hypothalamus; Pre, During and Post, pre-, during- and post-attack.

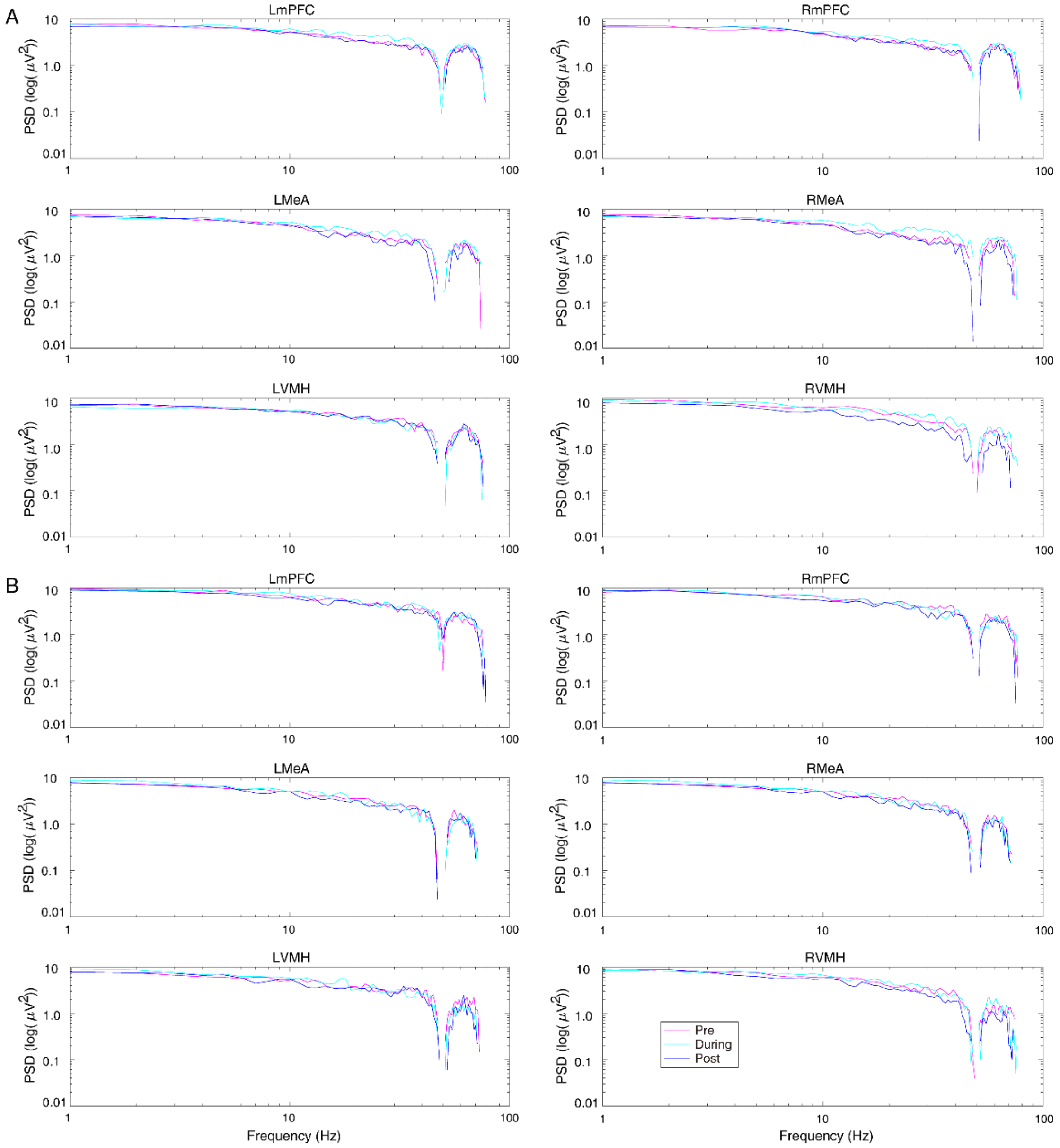

Figure S2. The average waveforms of power spectral density (PSD) for each brain area and each situation for the socially isolated mice (A) and the cohousing mice (B). For each subject, the power spectral densities were calculated for each segment, each brain region, each time condition (i.e. pre-, during- and post-attack). The power spectral densities were averaged between segments for each brain region, each time condition and each subject, then further averaged across all subjects for each group. Note that a notch filter to eliminate possible power line interference at 50 Hz during raw LFP

recording, and the raw LFP recordings were filtered offline using a bandpass filter of 0.5-80 Hz. LmPFC and RmPFC, the left and right medial prefrontal cortex; LMeA and RMeA, the left and right medial amygdala; LVMH and RVMH, the left and right ventromedial hypothalamus; Pre, During and Post, pre-, during- and post-attack.

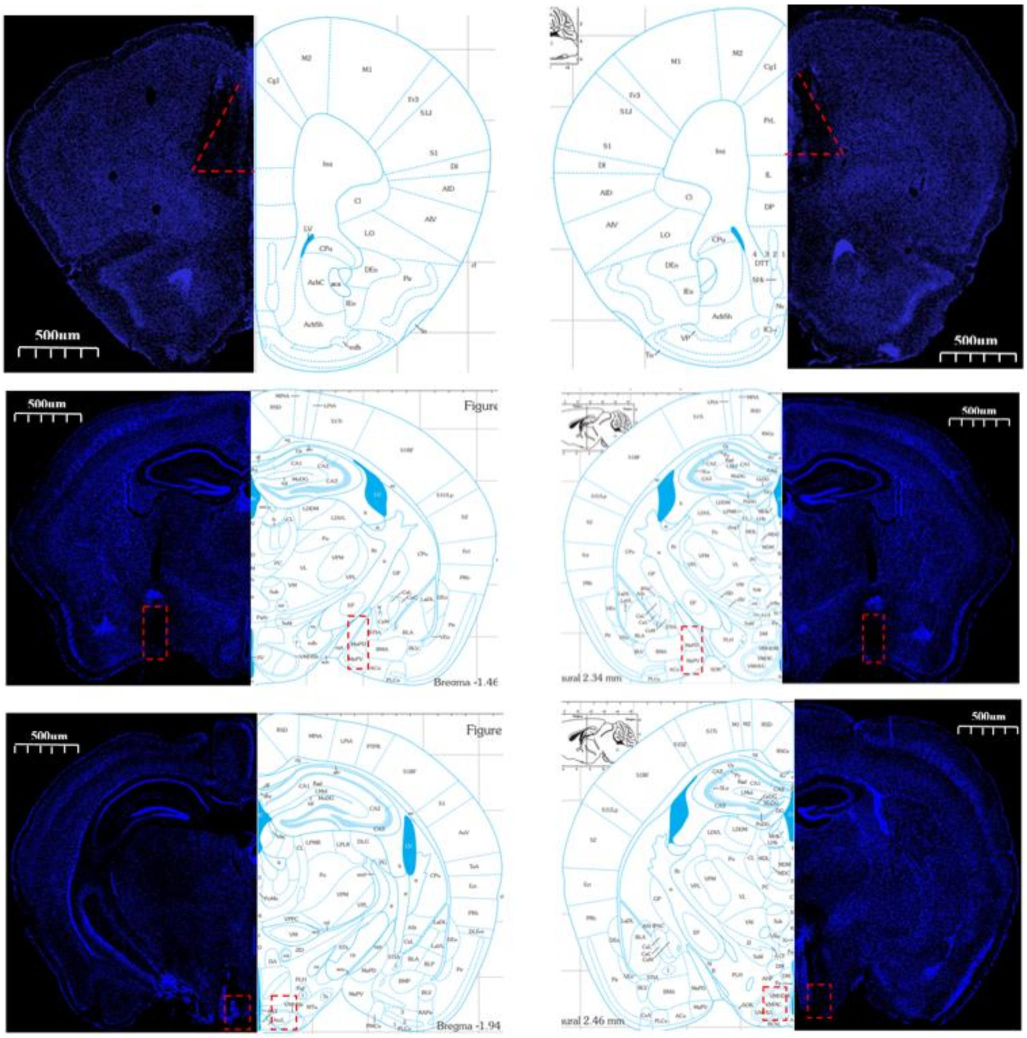

Figure S3 Verification results of electrode positions for both sides of the medial prefrontal cortex (the first row), medial amygdala (the second row), and ventromedial hypothalamus (the last row). Each rectangle drawn with

red dotted line represents one of above target areas.

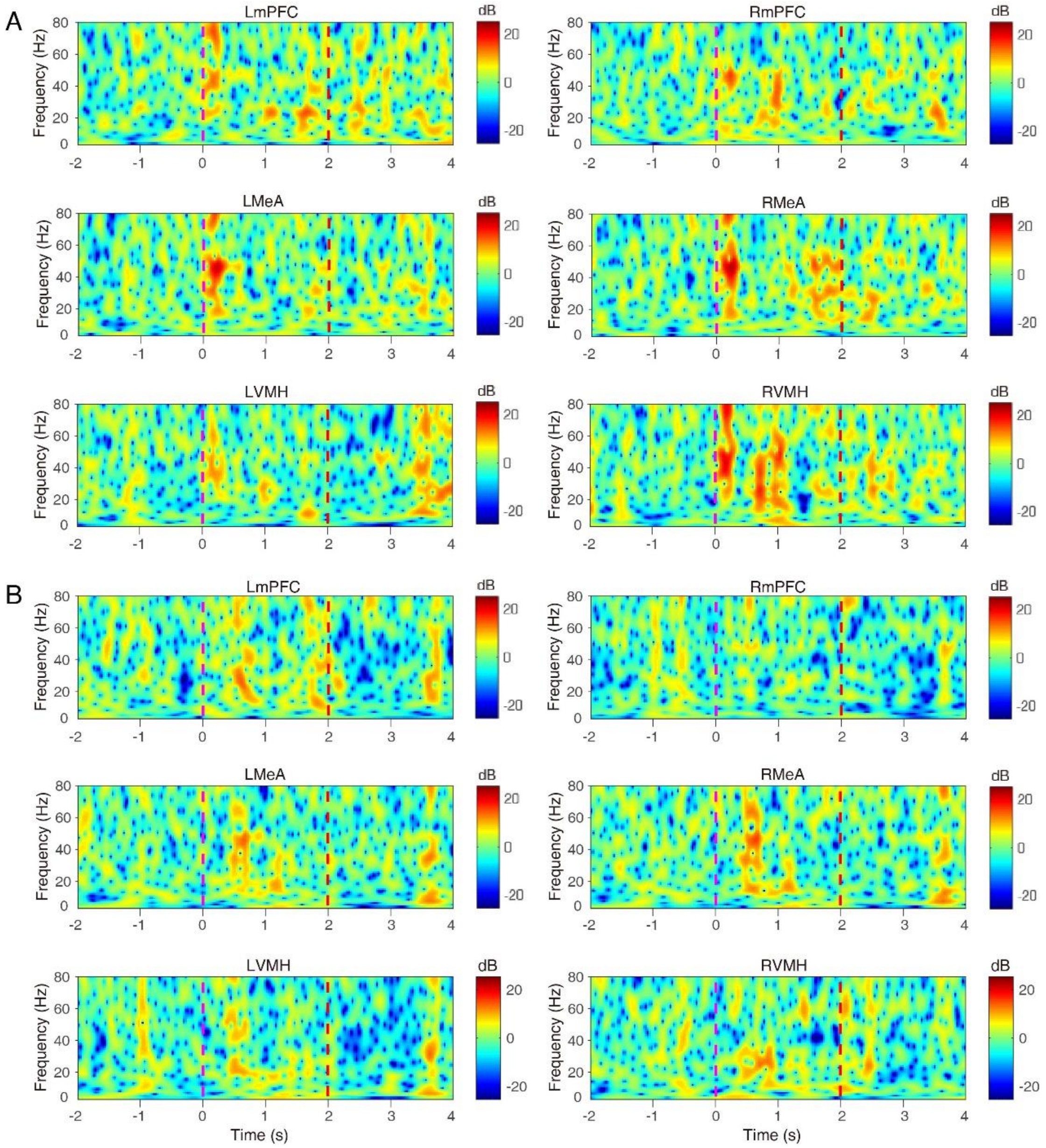

Figure S4. Time-frequency maps of grand mean local field potential (LFP) waveforms across subjects for each brain area and each situation for the socially isolated mice (A) and the cohousing mice (B). LmPFC and RmPFC, the left and right medial prefrontal cortex; LMeA and RMeA, the left and right medial amygdala; LVMH and RVMH, the left and right ventromedial

hypothalamus. The pink vertical dotted line denotes the onset of attack, while the red vertical dotted line denotes the ending of attack.
